# Supplementary material for: Structured transverse orbital angular momentum probed by a levitated optomechanical sensor
Source: Nat Commun. 2023 May 6;14:2638. doi: 10.1038/s41467-023-38261-7 (PMC10164142; doi:10.1038/s41467-023-38261-7)
Supplement: Supplementary file 1 — Supplementary Information [file 41467_2023_38261_MOESM1_ESM.pdf]

Supplementary Information

A. Simplified model for the origin of TOAM and torque

Consider the rod under illumination by the two beams (Fig.1 in the main text). As we have shown in the main text, a TOAM density is present in this illumination. This TOAM can be understood in very simple terms: opposite ends of the rod will be pushed (via an optical pressure force caused by each beam's orbital momentum density $\mathbf{P}$) in opposite directions, generating a torque on the particle.

We will simplify the situation with an approximate model by assuming that each volume element of the rod is subject to the illuminating field *only* (i.e., this model neglects the scattering from one part of the rod acting on another part). The *scattering force* acting on each volume element $\mathbf{f_{SF}}$ would then be proportional to the local momentum density vector of the illumination at each volume element $\mathbf{f_{SF}} \propto \mathbf{P}$ where $\mathbf{P} = \mathbf{P}^{\mathrm{e}} + \mathbf{P}^{\mathrm{m}}$. According to this approximation, the net force on the rod would be:

$$\mathbf{F} = \iiint_{\mathcal{V}} \mathbf{f_{SF}}\, \mathrm{d}V \propto \iiint_{\mathcal{V}} \mathbf{P}\, \mathrm{d}V,$$

which in this case would be zero due to the symmetry of the illumination pushing the top and bottom parts in opposite directions.

However, these balanced forces will exert a non-zero net torque on the rod, essentially by pushing the top and bottom of the rod in opposite directions. The mechanical torque caused by a force on an object, evaluated around the origin of coordinates, is defined as $\tau = \mathbf{r} \times \mathbf{f}$. The *net* torque, therefore, by integration of the torque on each volume element, and applying the same model above where $\mathbf{f_{SF}} \propto \mathbf{P}$, is given by:

$$\mathbf{N}_{\mathrm{model}} = \iiint_{\mathcal{V}} \mathbf{r} \times \mathbf{f}\, \mathrm{d}V \propto \iiint_{\mathcal{V}} \underbrace{\mathbf{r} \times \mathbf{P}}_{\mathbf{L}}\, \mathrm{d}V,$$

where the term $\mathbf{L}(\mathbf{r}) \equiv \mathbf{r} \times \mathbf{P}(\mathbf{r})$ is the *definition* of orbital angular momentum density of light, which in this case becomes transverse. By virtue of being misaligned with respect to the origin, each of the two beams creates a TOAM density at the volume occupied by the rod which can be integrated to estimate the direction of the torque. Interestingly, this explanation does not even require the presence of the transverse vortex arrays. In this model we also ignored the SAM density of light which can also cause a torque on the individual volume elements.

This model is great for gaining an intuitive understanding of the torque, but it is unsuitably crude when quantitatively assessing the torque. On one side, SAM should be included. On the other hand, the rod is big compared to the wavelength and it interacts strongly with the illumination. The scattering from one part greatly affects the force experienced by another part, and so the torque on the rod cannot be simply calculated as a volume integral of the OAM and SAM.

To take the full physics into account, in the main text we applied the Maxwell Stress Tensor formalism to calculate the torque from first principles, via conservation of angular momentum, using the full scattered fields from numerical simulations (Fig.2**d**).

B. Effect of beam offset

In this manuscript we repeatedly state that we have found a ”straightforward and robust” method for generating intrinsic TOAM. Here we justify this claim, by showing the effect of the beam separation on the generated orbital angular momentum, see Fig. S1. The existence of the TOAM does not depend on a critical beam separation. We note that the phase winding is non-linear, and depends on the magnitude of the separation. By $\delta = 1.0\ \mu$m the intensity standing wave is beginning to lose structure.

See further Supplementary Movie 1 for a video illustrating the generation of vortices when the beam separation is varied.

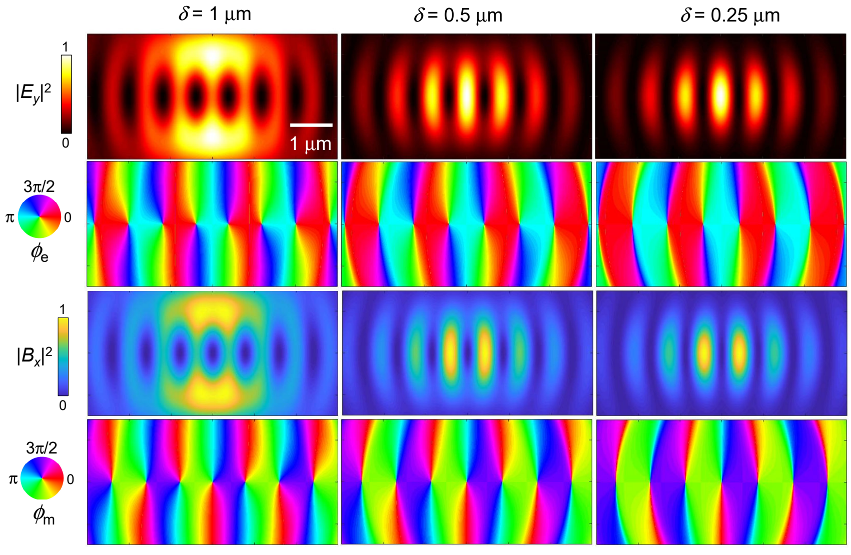

Fig. S1. **Variation in TOAM with beam offset**. From top-to-bottom the rows show the electric field intensity, transverse electric field phase distribution, magnetic field intensity, transverse magnetic field phase distribution. From left-to-right we consider beam separations of $\delta = 1.0$, 0.5, 0.25 μm.

C. Transverse spin angular momentum (SAM)

In order to optically trap the silicon nanorod, beam focusing is required. It is well-known that when a single linearly polarised collimated beam is focused, the polarisation at the focus becomes elliptically polarised in the transverse plane because of the generation of a longitudinal field with a phase difference with respect to the transverse field [54]. This transverse elliptical polarisation corresponds to a transverse spin and is associated with a transverse SAM. Since our optical trap uses two interfering focused beams, the nature of the transverse is complicated further. Fig. S2 shows the transverse spin density of the optical trap when $\delta_y = 0.5\,\mu$m, indicating the presence of transverse SAM.

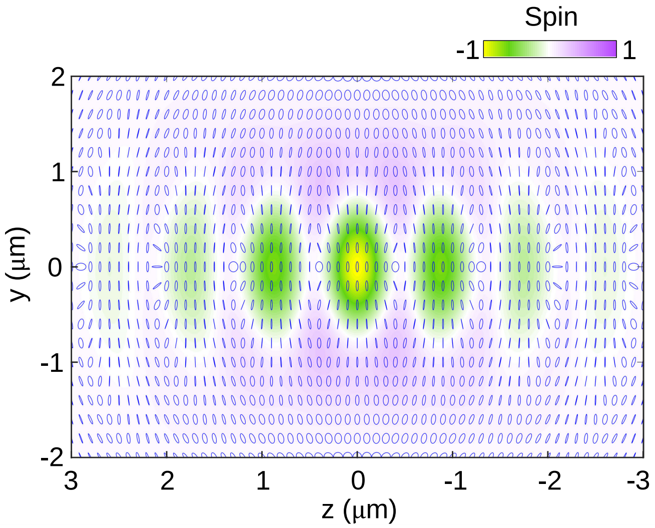

Fig. S2. **Transverse SAM**. Transverse spin S_x and normalised polarisation ellipses indicating that the points of close-to-circular polarisation are near electric field nodes.

D. OAM torque and SAM torque

The previous section showed the existence of a transverse SAM density in the centre of the optical trap, so there is both OAM and SAM in this system for the nanorod to experience a torque from. Optical torques occur when a body gains angular momentum from an optical field. Since optical angular momentum can be split into SAM and OAM, optical torques can similarly be split into torques that derive from SAM and OAM respectively. The Methods section of the main text describes how the total torque on the silicon nanorod is calculated with the Maxwell stress tensor approach, but Ref.[39] shows that the MST can be split into OAM and SAM terms. Using this torque decomposition, we split Fig.2**d** into OAM and SAM components. Fig. S3 shows this decomposition and at the origin where the total torque is strongest, the OAM torque is roughly 15 times larger than the SAM torque. The two torques also have opposite signs and so act against each other. We can therefore conclude that the OAM is the dominant source of torque in this optical trap.

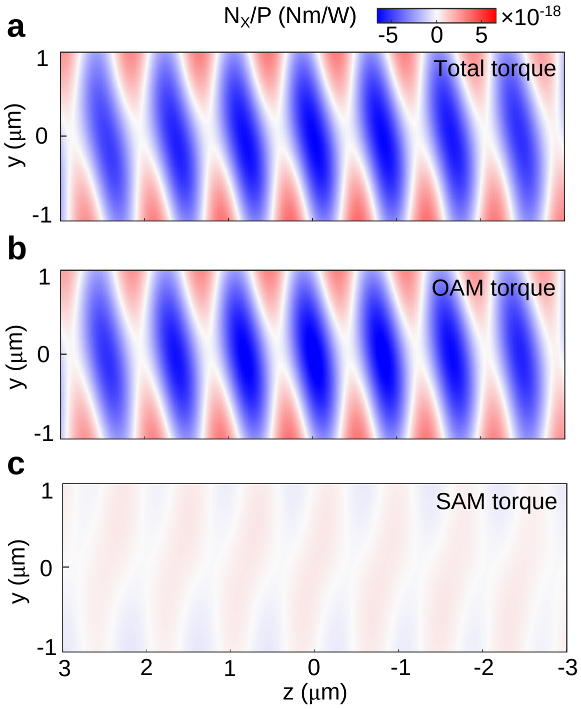

Fig. S3. **Torque decomposition**. **a** The torque map of the torque on the nanorod as the position of the nanorod is changed. Also shown in Fig.2**d**. **b** A map of the OAM part of the torque. **c** A map of the SAM part of the torque.

E. Longitudinal foci separation

Throughout the main text, the relative position of the two counter-propagating beams is defined by restraining $\delta_x = \delta_z = 0$ and varying δ_y. The δ_z separation can be difficult to quantify precisely in some experimental configurations so it can be instructive to investigate the δ_z dependence of the transverse optical torque in this optical trap, and what limitations it may present.

The intensity and phase of the electric field in the counter-propagating trap with $\delta_y = 500$ nm (roughly $\lambda/3$) is presented in Fig. S4**a-e**, but with different values for δ_z. As δ_z increases, the transverse phase vortices transition into regular standing wave mode structures with no significant change of phase along the y direction. This suggests that the TOAM present in the optical field is diminishing once the focal planes of the two beams are significantly far apart longitudinally. Such a result is expected given the nature of tightly focused beams and the rapid change in their fields as one moves away from the focus.

Building on these results, the δ_z dependence of the optical torque on the silicon nanorod probe can be simulated by inserting the particle's scattering into the incident fields and then using the Maxwell stress tensor approach. The torque is of greatest magnitude when $\delta_z = 0$ and the transverse vortices are most prevalent. Once the distance between the focal planes of the two beams ($2\delta_z$) is roughly an order of magnitude greater than the wavelength, the torque greatly diminishes. We also observe some mild asymmetry in the particle's response with respect to δ_z. The size of this constraint will in general depend on the degree of beam focusing and the specific of the trapping system. An order-of-magnitude estimation for our experimental δ_z can be generated by noting the beam divergence after the trapping lenses (beam diameter doubles after 5 m), the lens focal length (2.97 mm) and the lens diameter (4 mm). Simple ray optics can be used to find an experimental estimation of $\delta_z \approx 2\,\mu$m. We therefore conclude that our trapping system has a suitably small foci separation for generating an incident field with transverse phase vortices and generating a torque via TOAM.

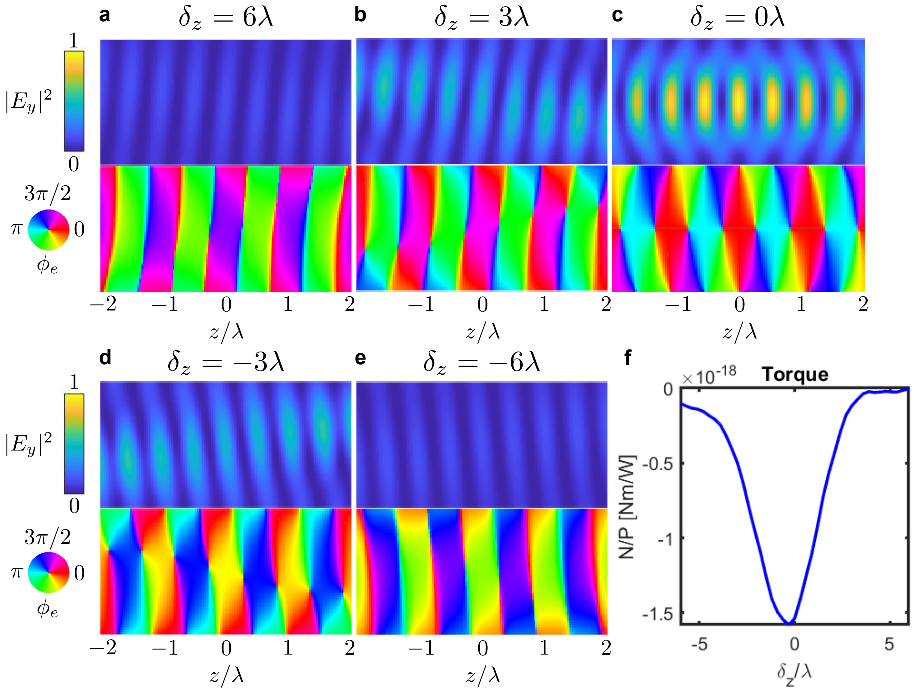

Fig. S4. **Effect of longitudinal foci separation**. **a-e** The intensity and phase of the electric field in a TOAM optical trap with various values for δ_z. **f** The optical torque on a silicon nanorod located at the origin orientated vertically varying with δ_z.

F. Effect of angle between the beams

As discussed in the Methods, our method for introducing a separation between the beams also introduces a small angle between them. Since we know the properties of our lenses, we know the angle between the beams, which is approximately 0.04 rad (2.5°) at the point of maximum torque. Fig. S5 shows the effect on the generated TOAM of an angle between the beams, clearly illustrating that this small angular misalignment does not qualitatively alter the angular momentum structure.

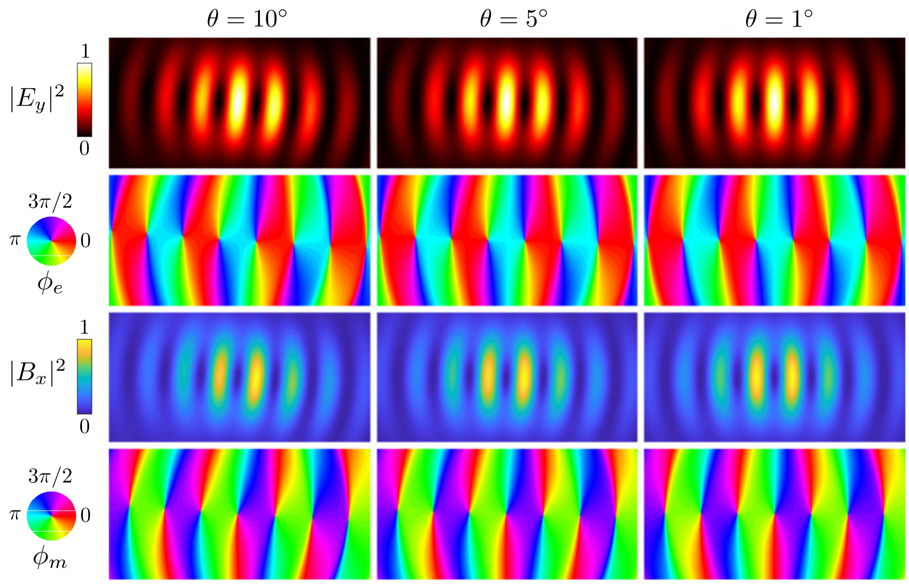

Fig. S5. **Variation in TOAM with an angle between the beams**. From top-to-bottom, the rows show the electric field intensity, transverse electric field phase distribution, magnetic field intensity, transverse magnetic field phase distribution. For a beam separation of $\delta_y = 0.5\,\mu$m, from left-to-right we consider an angle between the beams of $\theta = 10°, 5°, 1°$.
