# Supplementary material for: Structured transverse orbital angular momentum probed by a levitated optomechanical sensor
Source: Nat Commun. 2023 May 6;14:2638. doi: 10.1038/s41467-023-38261-7 (PMC10164142; doi:10.1038/s41467-023-38261-7)
Supplement: Supplementary file 3 — Description of Additional Supplementary Files [file 41467_2023_38261_MOESM3_ESM.pdf]

Description of Additional Supplementary Files

Supplementary Movie 1: Generated vortices with varied beam separation. Animated electric field $E_y(t) = \Re(E_y e^{-i\omega t})$ showing the two Gaussian beams, using identical parameters as Fig. 2 in the main text (λ = 1550 nm, w_0 = 1.0 μm), but with a δ parameter varying between 3.0 μm and 0.5 μm, and back. When the Gaussian beams are close together, we can see the array of phase vortices as points with circulating phase around them.
