# Supplementary figures and images for: Structured transverse orbital angular momentum probed by a levitated optomechanical sensor
Source: Nat Commun. 2023 May 6;14:2638. doi: 10.1038/s41467-023-38261-7 (PMC10164142; doi:10.1038/s41467-023-38261-7)

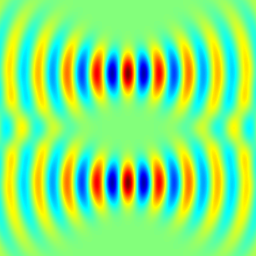

Supplement: Supplementary file 4 — Supplementary Movie 1 [file 41467_2023_38261_MOESM4_ESM.gif]
